# Kin and multilevel selection in social evolution: a never-ending controversy?

**DOI:** 10.12688/f1000research.8018.1

**Published:** 2016-04-28

**Authors:** Jos Kramer, Joël Meunier

**Affiliations:** 1 Zoological Institute, Evolutionary Biology, Johannes Gutenberg University Mainz, Mainz, Germany; 2 Institut de Recherche sur la Biologie de l’Insecte, UMR 7261, CNRS, Université François Rabelais, Tours, France

**Keywords:** cooperation, altruism, sociobiology, group selection, levels of selection, inclusive fitness

## Abstract

Kin selection and multilevel selection are two major frameworks in evolutionary biology that aim at explaining the evolution of social behaviors. However, the relationship between these two theories has been plagued by controversy for almost half a century and debates about their relevance and usefulness in explaining social evolution seem to rekindle at regular intervals. Here, we first provide a concise introduction into the kin selection and multilevel selection theories and shed light onto the roots of the controversy surrounding them. We then review two major aspects of the current debate: the presumed formal equivalency of the two theories and the question whether group selection can lead to group adaptation. We conclude by arguing that the two theories can offer complementary approaches to the study of social evolution: kin selection approaches usually focus on the identification of optimal phenotypes and thus on the endresult of a selection process, whereas multilevel selection approaches focus on the ongoing selection process itself. The two theories thus provide different perspectives that might be fruitfully combined to promote our understanding of the evolution in group-structured populations.

## Introduction

Why should an individual cooperate to benefit others? This question pinpoints one of the central theoretical problems of sociobiology ^1– 3^. Cooperative behaviors such as altruism (an action that benefits others at one’s own expense) would reduce the fitness of the performer relative to selfish individuals that do not perform the behavior, and hence should be selected against ^4– 6^. However, this expectation is in striking contrast to the ubiquity of cooperation in nature, which occurs among ‘simple’ microorganisms ^7, 8^ and within highly complex eusocial societies alike ^9^.

Kinship and group selection are two key concepts of modern sociobiology that have been proposed to help resolve this apparent conundrum ^1, 6^. Despite their common origin in the writings of Charles Darwin (cf. 10), the developments of these two concepts in the modern kin and multilevel (or group) selection theories followed diverging paths and fueled a persisting and often heated debate about their relevance and usefulness in the study of social evolution (e.g. 11– 13). Here, we provide a concise introduction to the two theories and the controversy surrounding them as well as highlight the complementarity of the approaches typically taken by their proponents. To this end, we first separately introduce the two theories and then point to the roots of the controversy. We subsequently review two important aspects of the current debate in more detail: the presumed formal equivalency of the two theories and the notion of group adaptation. We overall suggest that these issues illustrate the complementary nature of the perspectives offered by the kin selection and multilevel selection theories.

## Kin and multilevel selection theories in a nutshell

### Kin selection theory

**Figure 1.**
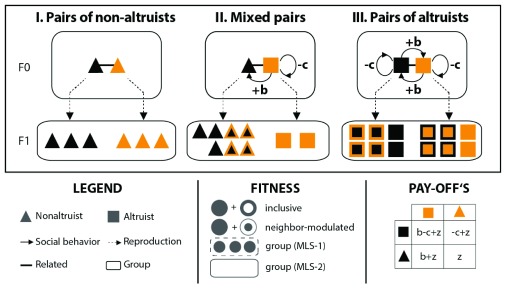
Social interactions in a group-structured population. In this example, non-altruists and altruists (symbolized by triangles and squares, respectively; see the *legend* for details) form (I) pairs of non-altruists, (II) mixed pairs, and (III) pairs of altruists. Altruists and non-altruists do not share the gene(s) causing the altruistic behavior but are overall related (i.e. genetically similar with respect to other traits). Upon reproduction, the (F1) progeny of each pair forms an independent group. Non-altruists do not express social behaviors and reproduce according to a baseline fitness (here arbitrarily fixed at z = 3). In contrast, altruists (unconditionally) confer a fitness benefit (b = 4) onto their partner at a cost (c = 1) to themselves. Although altruists incur a direct fitness cost, they benefit indirectly from assisting their partner and hence overall increase their inclusive fitness (see the *fitness box* for an illustration: inclusive fitness is composed of a direct [unicolored symbols] and an indirect [symbols framed in the same color] component). Similarly, their neighbor-modulated fitness is increased if they are assisted by their partner. However, altruists have a relative fitness disadvantage within mixed pairs because they increase the fitness of their non-altruistic partner at a cost to themselves and without receiving help in return (see *pay-offs box* for details). Hence, altruism can be disadvantageous even within groups of overall related individuals (precisely if they do not share the altruistic gene). In contrast, uniform pairs of altruists produce more offspring than mixed groups or groups of non-altruists (they hence have a higher group fitness in an MLS-1 framework; the group fitness in an MLS-2 framework corresponds to the number of groups produced and is here the same for all pairs). Positive assortment of altruists (e.g. due to limited dispersal) reinforces this ‘group-advantageous’ effect and can explain the evolution of cooperation in the long run.

Interacting organisms may have an evolutionary incentive to help each other (or at least to hurt each other less) if they share genes, and the magnitude of this incentive should increase with the degree of relatedness between them; this is the central tenet of William D. Hamilton’s *inclusive fitness theory* ^14– 16^ (the term *kin selection theory* was coined by John Maynard-Smith ^11^ and is here used as a synonym for ‘inclusive fitness theory’ to comply with its conventional use). This tenet is encapsulated in a very simple form in *Hamilton’s rule*, which states that a (gene for a) social behavior is favored by natural selection if *rb*- *c* > 0, where *c* is the fitness cost to the individual performing the behavior, *b* equals the fitness benefit to the recipient(s), and *r* is the genetic relatedness between them ^14, 15^. The rule thus formalizes the realization that natural selection acts not only through direct effects of a behavior on the actor’s own fitness (often measured as reproductive output) but also through indirect effects on the fitness of the actor’s relatives (that have an above-average probability of sharing the actor’s genes, including the one[s] that cause the social behavior in question) ^1^. Moreover, it provides a potential solution to the central problem of sociobiology, as it shows that even costly social behaviors can be favored by natural selection as long as the direct costs are outweighed by a sufficient amount of indirect benefit to sufficiently closely related individuals ( Figure 1) ^17^. Note, however, that the application of Hamilton’s rule—and thus kin selection theory—is not restricted to altruistic behaviors: *rb* and *-c* represent, respectively, the indirect and the direct fitness consequences of *any* character of interest and hence can both be positive, negative, or zero ^18^. Accordingly, they can also represent mutually beneficial (both fitness components positive), spiteful (both components negative), or selfish (direct component positive and indirect component zero or negative) behaviors.

Alongside Hamilton’s rule, the concept of *inclusive fitness* is the second central element of kin selection theory. An organism’s inclusive fitness is defined as the sum of its direct (Darwinian) and indirect fitness components ( Figure 1). The latter is calculated as the relatedness-weighed sum of those effects on the fitness of other individuals for which the organism is causally responsible ^14, 17^. Inclusive fitness is thus an actor-centric approach that examines how a focal individual influences its own fitness and that of its social partners ^19^. Hamilton ^14^ also suggested an alternative and (by now) increasingly used approach to account for direct and indirect fitness effects (e.g. 20– 22): the *neighbor-modulated fitness* (sometimes referred to as *personal* or *direct* fitness). The two approaches differ in how the indirect component is conceptualized: in contrast to inclusive fitness, neighbor-modulated fitness is a recipient-centric approach and thus examines how social partners influence the fitness of a focal individual ^19^ ( Figure 1). The two approaches are usually seen as equivalent, as they predict the same overall response to natural selection ^18^ (but cf. 23, 24). The inclusive fitness approach, however, comes with one significant conceptual advantage: in principle, an individual is causally responsible for both its direct and indirect fitness and hence can control its inclusive fitness. Thus, natural selection might favor organisms that act as if they are attempting to maximize their inclusive fitness ^14, 25– 27^ (but cf. 28, 29). The possibility of conceptualizing individuals as maximizing agents (an optimality approach ^30^) greatly facilitates the linking of theoretical and empirical research and has been central to the study of adaptation in behavioral and evolutionary ecology ^19^.

Over the last five decades, kin selection theory has been extensively developed and generalized (e.g. 20, 31– 35; see 22, 23 for book-length treatments) beyond the limited scope of Hamilton’s original formalization ^14– 16^. For instance, Hamilton originally defined relatedness as a genealogical measure of shared ancestry ^14^ but quickly realized that this is only one (albeit by far the most frequent) way of generating the above-average genetic similarity among individuals that ultimately drives the evolution of many cooperative behaviors ^16, 31, 36^. In contemporary discussions, relatedness is accordingly more broadly defined (e.g. 23, 24) and encompasses any genetic similarity, regardless of whether it arose by common descent or by other means such as green-beard effects ^37, 38^. Moreover, the generalization of kin selection theory resulted in the development of general versions of Hamilton’s rule (most notably on the basis of the Price equation; see below and 34) that—unlike the original version—make no assumptions such as weak selection or additivity of fitness payoffs (see also 18, 39, 40). In these general versions, the cost-benefit parameters no longer denote simple fitness payoffs of social interactions but rather partial regression coefficients that quantify the overall statistical association among an organism’s phenotype/genotype, its fitness, and the phenotype/genotype of its social partners ^17, 41^. Concomitant with its generalization, kin selection theory has become the dominating framework for the explanation of social behavior from an evolutionary viewpoint (e.g. 42, 43). Its most prominent empirical prediction, namely that social behavior should correlate with relatedness, has been supported across diverse taxa (the equally important impact of the costs and benefits received less attention, but see 44, 45), and kin selection theory has greatly contributed to our current understanding of a variety of biological phenomena such as dispersal, reproductive skew, and queen-worker conflicts in eusocial insects ^46, 47^.

### Multilevel selection theory

The central tenet of multilevel (or group) selection theory conveys that selection not only acts on individuals but can act (simultaneously) on multiple levels of biological organization, including cells and/or groups ^48^. This view suggests that even if behaviors that benefit other individuals are selectively disadvantageous at the level of the individual, they might still evolve if they are advantageous at—and hence selected for on—a higher level of the biological hierarchy (e.g. on the group or colony level) ^6, 48^. Altruism, for instance, is costly for the altruistic individual, but groups containing a higher proportion of altruistic individuals usually have a competitive advantage over groups that are composed mostly of selfish individuals (e.g. because altruistic groups are more productive or superior in direct confrontations). In such situations, altruism can evolve—driven by a process of selection *between* groups—even against the background of selection favoring selfishness *within* each group (e.g. 49, 50). This is the potential solution of the central problem of sociobiology from a multilevel perspective ( Figure 1). The applicability of multilevel selection theory, however, is not limited to situations in which selection pressures on different hierarchical levels are opposing. Multilevel selection approaches more generally examine the direction and strength of (naturally occurring or experimentally applied) selection pressures on multiple hierarchical levels, investigate the mediators (e.g. indirect genetic effects ^51– 53^) and magnitude of their respective contributions to total evolutionary change ^50, 54, 55^, and explore effects of selection on group traits ^56^.

Interestingly, there is currently no unanimously accepted formal theory of multilevel selection (e.g. 57– 60) apart from the broad consent that defines group selection as natural selection based on the differential survival and reproduction of groups ^6, 51, 61^ (but cf. 54). As a result, both individuals and groups can be found as focal units of attention ( Figure 1) in multilevel selection approaches. Similarly, the definitions of *group fitness* and *group* vary among studies depending on whether they aimed at explaining the changing frequency of different types of *organisms* in a group-structured population or at explaining the changing frequency of different types of *group* in a meta-population of groups. In the first case, group fitness is often defined as the average (or total) fitness of its constituent organism (multilevel-selection-1, or *MLS-1*; Figure 1 ^62– 64^), and the group as a set of interacting individuals that influence each other’s fitness, but not the fitness of individuals outside the group, with respect to a particular trait ( *trait-groups* ^49, 61^; see 48 for a discussion of this concept). In the second case, group fitness is defined as the expected number of offspring groups ( *MLS-2*; individual fitness is then usually defined separately and on a different timescale ^62– 64^). MLS-2 approaches often explicitly incorporate group-level events such as fission or extinction and thus assume that groups of individuals undergoing such group-level events can be identified in the population ^64^. Groups are consequently more narrowly defined as geographically discrete, multigenerational, and reproductively isolated demes ( Figure 1) ^48^.

It is important to note that MLS-1 and MLS-2 approaches are not equivalent, as they relate to different natural processes ^48^ (but cf. 58). In an MLS-1 scenario, groups ‘merely’ generate the population structure that affects the fitness of *individuals*. Hence, groups can propagate by producing individuals as long as these individuals form groups themselves at some stage of their life (e.g. after blending in a common mating pool). In contrast, groups need to reproduce in an ordinary sense in an MLS-2 scenario (i.e. by producing more groups) ^48^. Though sometimes seen as fostering confusion (e.g. 58, 65), the difference between MLS-1 and MLS-2 also provides an intriguing diachronic perspective on ‘major transitions’ in evolution—and thus on the evolution of the biological hierarchy itself—as such transitions involve a temporal shift from MLS-1 (groups *of* individuals) to MLS-2 (groups *as* individuals) ^66– 69^.

Despite its controversy-plagued history (see below), the multilevel selection theory has undergone a resurgence of interest in recent years (e.g. 6, 12, 58, 70– 72; see 48 for a book-length treatment). This is because it has provided novel perspectives on a variety of issues such as parasitic virulence ^73^, cultural group selection in humans (that is often envisioned as the outcome of warlike confrontation) ^74, 75^, or the ‘major transitions’ in evolution ^48, 69^.

## The controversy

### A brief history

The controversy surrounding the theories of kin and multilevel selection has a long and turbulent history (detailed, e.g., in 1, 24, 48, 61, 76). Until the second half of the last century, many biologists did not clearly distinguish between different levels of selection, and it was often uncritically assumed that group selection would easily prevail over individual selection (e.g. 77) or that individual selection alone would foster adaptations ‘for the good of the group’ (e.g. 78). It was the rebuttal of these ‘naïve’ assumptions (though not of the theoretical plausibility of group-level thinking *per se*; cf. 6) that widely led to the rejection of group selection as a significant evolutionary force ^79^. Notably, kin selection theory—along with other theoretical frameworks such as evolutionary game theory ^80^ and selfish gene theory ^37, 81^—was initially developed as an alternative to group selection (e.g. 11, 15), which likely contributed to an increasing polarization in disfavor of arguments based on group-level thinking (e.g. 37).

However, the demise of group selection was only temporary. Subsequent studies dropped the ‘naïve’ assumption of the unconditional superiority of group selection and instead acknowledged that selection within groups often undermines selection among groups (e.g. 49, 82– 84). Building on this premise, trait-group models suggested that group selection can drive evolutionary change even when opposed by within-group selection and that a periodical blending of groups (e.g. in a common mating pool) can prevent the seemingly inevitable fixation of selfish types within groups ^49, 84^. Moreover, empirical studies demonstrated that experimentally applied group selection can drive evolutionary change (e.g. 85– 87; reviewed in 59, 88) and argued that early models had restricted the applicability of group selection by deploying unrealistic assumptions, such as the notion that group and individual selection are always diametrically opposed (reviewed in 6, 51). Interestingly, Hamilton himself showed that multilevel selection was formally equivalent with his theory of inclusive fitness ( 31, see next section), suggesting that the two theories simply outline different perspectives on the same natural processes. In some minds, this realization closed the debate, as the choice between the two theories seemed to have become a mere matter of personal taste. But far from it, the relationship between kin and multilevel selection remained controversial.

Over the last four decades, the group selection controversy has lost little of its initial momentum and continues to polarize opinions fueled by semantic debates (reviewed in 12, 65, 70) and, ultimately, the different implications the two theories seem to have for the evolution and self-perception of our own species ^89– 91^ (see also 92 and associated responses). Accordingly, some biologists contest the usefulness of multilevel selection in the study of social evolution in general ^12, 65^, whereas others call for a reframing of the theoretical foundations of sociobiology from a multilevel perspective ^6^. Nevertheless, the focus of the controversy has shifted away from the question of whether group selection occurs at all and now mainly revolves around (the consequences of) its presumed formal equivalence with kin selection theory and the question of whether group selection can lead to (group) adaptation. These are the two topics we will discuss below.

### Kin and multilevel selection: formally equivalent theories?

Most biologists consider kin and multilevel selection formally equivalent (e.g. 24, 40, 61, 93, 94), but this view is not universally accepted and the number of dissenting voices has recently grown (e.g. 95– 102). What, then, is the basis for the formal equivalency of the two approaches, and why is it still controversial?

On a practical level, the compatibility of kin and multilevel selection relies on the fact that both theories require positive assortment of (genetically) similar individuals for cooperative behaviors to evolve ( 31, 103, 104; see 105– 108 for examples). From a multilevel perspective, positive assortment increases the scope for between-group selection, as it will make groups internally more homogeneous and thus reduce the potential for within-group selection ( Figure 1) ^48^. From a kin selection perspective, positive assortment ensures that costly social behaviors such as altruism are preferentially directed toward individuals that show the behavior themselves ^31^. This directionality is crucial: altruistic traits are selectively disadvantageous even when directed at otherwise (genetically) very similar non-altruists ( Figure 1) and thus can evolve only if altruists sufficiently often interact with other altruists, thereby increasing their *average* inclusive fitness over that of non-altruists ^31^. In practical terms, the compatibility of kin and multilevel selection hence conveys that individuals expressing social behaviors (such as altruism) have a higher inclusive fitness than selfish individuals, whenever selection between groups is stronger than selection within them, and *vice versa* ^109^.

On a theoretical level, the compatibility of kin and multilevel selection is conventionally understood to predicate that group selection models can *always* be recast in terms of inclusive fitness ^39, 93^. This formal equivalency is usually inferred by using an equation developed by George C. Price ^110, 111^ that expresses the intergenerational, population-level response to natural selection in a heritable trait as the covariance, taken over all individuals within the population, between an individual’s trait and its fitness (here measured as its fecundity; e.g. 31, 40, 94). The Price equation allows partitioning the evolutionary change into its direct and indirect components and can be used to derive Hamilton’s rule (the kin selection approach; ^18, 22, 34, 36^). However, it also lends itself to partition the evolutionary change into effects at the individual and group levels ^22, 31, 40, 48, 94, 111^. Hence, kin and multilevel selection are formally equivalent when formulated as alternative decompositions of the Price equation, as both approaches make it possible to correctly compute the total evolutionary change. The approaches differ merely in how this change is partitioned and thus offer different, potentially complementary ways of viewing evolution in structured populations ^31, 40, 94, 112^. Two points, however, deserve a closer examination: firstly, the above-described decomposition of multilevel selection applies only to scenarios of the MLS-1 type (as group fitness is defined as average individual-level fitness of group members; ^48^). Secondly, the multilevel partitioning requires individuals to be nested in non-overlapping groups; the kin selection approach comes with no such requirement and thus is arguably more general within the Price framework ^24, 31, 48^.

Albeit most commonly used in theoretical studies, the Price framework is not the only approach to study multilevel selection. An alternative approach (that is often adopted in empirical studies; e.g. 55, 113, 114) is offered by contextual analysis ^48, 50, 115^. Like the Price equation, contextual analysis partitions the change due to natural selection (in MLS-1 scenarios; ^48^) into individual and group effects; but unlike the Price equation, it detects group selection only if group effects on fitness remain even after controlling for individual effects. Contextual analysis thus accommodates a classic criticism against the multilevel partition of the Price equation, which can detect a component of between-group selection even in non-social contexts (i.e. when the evolution of the population can be predicted without taking group structure into account), and hence might not always accurately reflect the true causal effect of group selection ^48, 115^. However, contextual analysis has problems of its own, as it detects group selection when there is no variation in fitness among groups (e.g. because they all have the same productivity), but individual fitness depends on their ranking within the group (soft selection; ^48, 50, 115^; but cf. 59). Owing to these problems, it is still controversial which approach is better suited to study multilevel selection (e.g. 48, 58– 60, 116, 117; see also 99, 118, 119). Note however that contextual analysis is formally very similar to modern kin selection models that are based on neighbor-modulated fitness. This supports the conjecture of an equivalency of kin and multilevel selection, as the two approaches seem inter-translatable even when multilevel selection is studied by using contextual analysis rather than the Price equation ^1, 30^.

Though suggested by both the Price equation and contextual analysis, the formal equivalency of kin and multilevel selection remains controversial (e.g. 95– 99). Most critics seem to reject (aspects of) the generalization of kin selection theory and instead contrast specific, narrowly defined formulations of kin selection with more general approaches to multilevel selection (e.g. 96, 99, 100, 102). For example, Wilson and Hölldobler ^100^ rejected the broad definition of relatedness, arguing that it leads to a departure from the earlier and heuristically very useful narrow definition of kin selection. As a consequence, it can be argued that kin selection is only a special case of multilevel selection because relatedness (i.e. genetic similarity) can occur without strict kinship and hence evolution can occur by group selection in the absence of selection among narrow-sense kin (for instance, if group selection acts on green beards) ^1^. Similarly, Van Veelen and colleagues ^96, 99^ (see also 95, 97) rejected attempts to generalize kin selection and then contrasted multilevel selection with a specific (rather than a general) version of kin selection in which the cost-benefit parameters of Hamilton’s rule denoted fitness payoffs (rather than partial regression coefficients). They showed that this kin selection approach can lead to incorrect predictions if the payoffs are non-additive (see also 120) and hence concluded that multilevel and kin selection are not equivalent. However, they compared a specific formulation of kin selection with their general formulation of multilevel selection and hence arguably could not refute assertions of the equivalency of the two theories that are based on general formulations of kin selection (cf. 17).

Interestingly, these rejections of the general formulation of kin selection ^99, 121^ (see also 122, 123) relate to a more extensive debate that was initiated by a high-profile charge of Nowak, Tarnita, and Wilson against the value of inclusive fitness theory in explaining the evolution of eusociality ^101^. This partly philosophical debate revolves around the question of whether the cost-benefit parameters in general formulations of Hamilton’s rule allow a causal interpretation at all. As these parameters denote partial regression coefficients, they can depend on relatedness ^124^ and population gene frequency, which can, for instance, lead to the counterintuitive result that a social behavior satisfies Hamilton’s rule at a low, but not at a high, frequency ^13^. Whereas critics consequently deny the general formulations of Hamilton’s rule any explanatory power and claim that they cannot accurately describe the evolutionary dynamics of any given system (e.g. 13, 101, 121, 125), others argue that they serve as a unifying principle that provides a super-ordinated framework for interpreting the results of otherwise disparate models in a general terminology (e.g. 18, 39). Overall, this debate reveals that the formal equivalency of kin and multilevel selection (somewhat obviously) holds only if equally general formulations of the two theories are pitted against each other and that the issue at the heart of the debate really is the question of whether such general formulations make sense from a heuristic perspective (e.g. 13, 18, 19, 101, 125– 128; see 17, 41 for in-depth reviews). Indeed, the question of whether and when general versions of Hamilton’s rule (and thus kin selection theory) provide a better/worse *causal* (rather than statistical) representation of the evolutionary process than the corresponding general approaches to multilevel selection (see also Conclusions section) might provide a fruitful avenue for future discussions.

Another line of reasoning against the equivalency of kin and multilevel selection suggests that even generalized formulations of kin selection cannot account for the long-term effects of events on the group level ^57, 95, 98^. Group-level events such as fission, fusion, or extinction often occur asynchronously, and Simon and colleagues ^57^ recently suggested that group selection should consequently be thought of (and analyzed) as an asynchronous, continuous-time process that is shaped by the combined, long-term effects of such group-level events. On this basis, they argued that although kin and multilevel selection are often equivalent when only one time interval between two group-level events is analyzed, they would almost never be equivalent in a dynamical setting because kin selection approaches could not account for the asynchronous nature of the group-level events ^57, 98^ (see also 129). Interestingly, they also suggest that the long-standing disagreement over the equivalency of kin and multilevel selection is based on oversimplified models of multilevel population dynamics and an inappropriate definition (via the Price equation) of group selection ^57, 98^: in its usual form, the Price equation traces the evolutionary change only over short periods (see above), assumes that all relevant processes such as reproduction or mass dispersion occur at a discrete set of time points, and is restricted to MLS-1 scenarios in which group-level events (other than mass dispersion) do not feature explicitly ^57, 98^. Thus, the Price equation (and contextual analysis) might be insufficient to capture all relevant aspects of the selection among groups.

Overall, the last word on the equivalency of kin and multilevel selection has surely not been spoken, as the partly philosophical character of the debate prevents it from being settled by theoretical or empirical results alone (cf. ^17^). However, even if it would turn out that the equivalency is untenable in some situations, kin and multilevel selection will surely continue to occupy largely overlapping domains, leaving evolutionary biologists with both the blessing and the curse of the existence of multiple theoretical frameworks to study social behavior.

### Does group selection lead to group adaptation?

One fundamental issue that triggered the initial rejection of group selection was the (then naïvely alleged) claim that it can foster group adaptation (i.e. promote the evolution of traits ‘for the good of the group’) ^6^. Although an evolutionary response to *group selection* has by now been demonstrated in a variety of laboratory and field studies (e.g. 85, 130– 132), the claim that it can foster *group adaptation* (or any adaptations at all) remains highly controversial ^12, 65^.

Recently, Pruitt and Goodnight ^56^ reported that natural colonies of the social spider *Anelosimus studiosus* are characterized by a site-specific mixture of ‘docile’ and ‘aggressive’ individuals and showed that experimentally constructed colonies with compositions mimicking the naturally occurring mixtures survived in the field but that colonies with deviating compositions perished. Experimental colonies with a perturbed composition that had survived at a ‘foreign’ site had shifted their composition toward a mixture that would have been optimal at their native site rather than toward the locally optimal mixture. Considering these results, Pruitt and Goodnight suggested that the composition of colonies differs between sites because of site-specific group selection and—as it is optimized to promote long-term colony survival at the native site—constitutes a group adaptation ^56^. This latter conclusion, however, did not go unchallenged ^133– 135^. Grinsted and colleagues ^134^ criticized that individual-level selection was not ruled out as an alternative explanation of Pruitt and Goodnight’s results, and Smallegange and Egas ^133^ indeed developed an environmental threshold model to explain Pruitt and Goodnight’s observations at the individual rather than the group level. Finally, Gardner ^135^ argued that colony composition is unlikely to maximize colony fitness and thus rejected the claim that the site specificity of colony composition constitutes a group adaptation.

Do the results of Pruitt and Goodnight ^56^ thus provide no evidence for a group-level adaptation after all? The answer depends on the definition of ‘group adaptation’. In a kin selection framework, adaptations are regarded as occurring at the level of the individual organism and to maximize an individual’s inclusive fitness ^19, 135, 136^. By analogy, group adaptation is thus understood as a process that is driven by between-group selection and optimizes phenotypes for the purpose of group fitness maximization ^135, 136^ (see also 137). This optimization process, however, is typically compromised by within-group selection because of conflicts among group members and thus will be favored by natural selection only if these conflicts are either absent (as for instance in clonal groups) or completely suppressed ^136^ (but cf. 138, 139), for example, through mechanisms such as fair meiosis ^140, 141^ or worker policing ^140, 142^. Accordingly, group adaptations are expected to occur only rarely in nature, where their demonstration would require showing that within-group conflict is absent and group fitness is maximized ^135, 136^. Pruitt and Goodnight ^56^ did not assess within-colony conflict and thus arguably provide no conclusive evidence for group adaptation according to the above definition—a view that is embraced by all critics of their interpretation ^133– 135^.

However, a different approach to group (and individual) adaptation is conventionally taken in a multilevel selection framework (e.g. 109, 143, 144). In accordance with the kin selection framework, a process would be defined as group adaptation if the trait frequency evolves toward (or has settled down at) the group optimum (i.e. the trait frequency that is predicted to evolve when only between-group selection is at work). Likewise, a process would be defined as individual adaptation in both frameworks if the trait frequency is driven by within-group selection only and hence evolves toward (or has settled down at) the individual optimum. However, differences between the kin and multilevel selection frameworks emerge if within- and between-group selection are aligned or if the metapopulation evolves toward (or has settled down at) a *compromise* (i.e. an intermediate trait frequency) ^61, 109^ (see also 1). In these situations, the (outcome of the) process would be called an individual adaptation in the kin selection framework, where adaptations are generally considered to occur at the level of the individual *irrespectively* of the strength of between-group selection ^136, 145^. In contrast, such compromises are often considered group adaptations in a multilevel selection framework, especially if they are (predominantly) driven by between-group selection ^109, 144^. Accordingly, while mechanisms of conflict suppression such as policing and punishment are a prerequisite of group adaptation in the kin selection framework, they are often considered group adaptations themselves in a multilevel selection framework ^6, 84^. In a reply to their critics, Pruitt and Goodnight adhere to this latter view and argue that the group-level trait ‘colony composition’ is shaped by site-specific group selection and hence constitutes a group adaptation ^143^.

This recent debate about the implications of Pruitt and Goodnight’s findings ^56^ sheds light on a clear distinction between the kin and multilevel selection frameworks. The kin selection approach typically grants the individual priority as an evolutionary agent because it appears as an adaptive unit and consequently allows a clear-cut distinction between individual and group adaptations even if selection acts on both the within- and between-group levels ^136, 145^ (but cf. 1, 61, 109). In contrast, the multilevel selection approach allows such a clear distinction only in special cases (namely if selection acts only on one level) but places more emphasis on the fact that the realized frequency of a social trait is usually a compromise of different selection pressures ^109, 143^. Moreover, the multilevel selection approach allows for situations in which selection pressures on the individual and group levels are (at least to some extent) aligned. This might be the case where collective traits (e.g. the superstructure of waterproof rafts built by fire ants through self-assembly ^146^) simultaneously promote the survival of the group and directly benefit the individuals within it. We suggest that both approaches may provide important insights into our understanding of social evolution and that, instead of focusing on their (semantic) differences, a more fruitful approach to the adaptiveness of social groups might be to ask how well adapted a particular social group is relative to the (theoretical) ideal of a conflict-free group ^1^ (see also 136, 147). Moreover, it is important to note that although the kin selection approach to group adaptation is more restrictive than the multilevel selection approach, both *in principle* allow for group adaptation. Thus far, the *formal* demonstration of group adaptation (i.e. a group-optimal and conflict-free outcome) according to Gardner and Grafen’s kin selection-based definition ^136^ is still pending. However, elaborate group-level traits such as the dance-language of honey bees ^148^ are good candidates that might live up to the definition of group adaptation within both frameworks.

## Conclusions

Kin and multilevel selection are two key concepts of modern sociobiology that provide different perspectives on the evolution of social behaviors. Unfortunately, these approaches are often pitted against each other in a seemingly endless (and largely semantic) debate that arguably impedes scientific progress ^91^ and prevents the benefits of the different perspectives from being harnessed.

Most biologists prefer kin selection over multilevel selection approaches as a matter of habit or personal taste ^12, 65^, and this preference seems partly justified as kin selection approaches have received more theoretical attention (and are hence highly versatile) ^20, 23^ and have been put to work in more empirical applications ^17, 46, 149^ (but note that the widespread acceptance of their formal equivalency implicates that empirical evidence for one theory cannot be used as evidence against the other). However, we believe that a bipartisan view on the kin and multilevel selection theories might ultimately prove more fruitful. After all, there might be situations in which one approach provides a more accurate representation of the causal structure of social interactions despite their (presumed) equivalency as statistical decompositions of evolutionary change ^17, 150^. For example, it might be more *causally apt* to describe the selection pressures on a segregation distorter allele that has negative effects on the fitness of its bearer in terms of multilevel selection (i.e. as opposing selection pressures at the gene and individual levels). Conversely, it might be more *causally* apt to describe the selection on cooperative behavior in pairwise interactions between related individuals in terms of kin selection, especially if those pairs are ephemeral and form only for the duration of the social interactions ^17^. More generally, kin selection might provide a more accurate representation of the causal structure of social interactions than multilevel selection where fitness pertains to individuals in the first instance (and group fitness is a simple function of the fitness of its constituent individuals; see also 22, 48), whereas the opposite might be true where fitness pertains to the whole group in the first instance (and individual fitness is determined by group fitness) ^150^. Such considerations of causal aptness might help to explain why multilevel selection was readily accepted for the study of major transitions ^48, 69^ but only slowly establishes itself in the field of behavioral ecology, in which social interactions are often studied in ephemeral ‘groups’ of genealogical kin ^42^. It is noteworthy that considerations of causal aptness seem less clear in the case of eusocial systems (such as ants, termites, and some species of bees and wasps; cf. 48) and that exactly these systems take center stage in the controversy surrounding the kin and multilevel selection theories (e.g. 100, 101, 126, 127, 151).

Interestingly, kin and multilevel selection approaches might ultimately prove to be very useful exactly when applied to the same system. Kin selection analyses often follow an optimality (‘adaptationist’) approach and accordingly try to identify the phenotype(s) with the highest overall fitness to extrapolate where a population will eventually stabilize ^18, 30^. The strength of kin selection (as a driver of evolutionary change over the course of one or multiple generations), however, is rarely reported ^30^. In contrast, multilevel selection approaches often follow an ‘evolutionary change’ approach and examine how a population will change its current configuration (for example, depending on the strength of within- and between-group selection or in response to an applied selection pressure) ^30, 59^. The optimal phenotype, however, is typically not identified in multilevel selection studies ^30^. The two perspectives opened up by the kin and multilevel selection approach, respectively, seem to be highly complementary ^30, 59^. Experimental studies already began to harness the translatability of the two approaches (e.g. 107, 108, 152, 153). However, we still eagerly await studies that make use of the full potential of their complementarity by combining both the ‘evolutionary change’ and ‘adaptationist’ perspective and the methods that come along with them. We believe that such studies would go a long way toward gaining a deeper understanding of the processes that ultimately drive the evolution of social behaviors in structured populations.
